# Identifying market risk for substandard and falsified medicines: an analytic framework based on qualitative research in China, Indonesia, Turkey and Romania

**DOI:** 10.12688/wellcomeopenres.15236.1

**Published:** 2019-04-16

**Authors:** Elizabeth Pisani, Adina-Loredana Nistor, Amalia Hasnida, Koray Parmaksiz, Jingying Xu, Maarten Oliver Kok

**Affiliations:** 1 Policy Institute, King's College London, London, London, WC2B 6LE, UK; 2 Faculty of Epidemiology and Population Health, London School of Hygiene & Tropical Medicine, London, London, WC1E 7HT, UK; 3 School of Health Policy & Management Health Care Governance, Erasmus University Rotterdam, Rotterdam, 3062 PA, The Netherlands; 4 Faculty of Criminal Law and Criminology, University of Groningen, Groningen, The Netherlands; 5 Migunani Research Institute, Sleman, Yogyakarta, Indonesia; 6 Department of Health Sciences, Vrije Universiteit Amsterdam, Amsterdam, The Netherlands

**Keywords:** falsified medicine, substandard medicine, counterifeit medicine, medicine quality, procurement, LMIC

## Abstract

**Introduction:** Substandard and falsified medicines undermine health systems. We sought to unravel the political and economic factors which drive the production of these products, and to explain how they reach patients.

**Methods:** We conducted in-depth case studies in China, Indonesia, Turkey and Romania. We reviewed academic papers and press reports (n = 840), developing semi-structured questionnaires. We interviewed regulators, policy-makers, pharmaceutical manufacturers, physicians, pharmacists, patients and academics (n=88). We coded data using NVivo software, and developed an analytic framework to assess national risks for substandard and falsified medicines. We tested the framework against cases reported to the World Health Organization, from countries at all income levels.

**Results:** We found that increasing political commitment to provision of universal health coverage has led to public procurement policies aimed at lowering prices of medical products. In response, legitimate, profit-driven pharmaceutical companies protect their margins by cutting costs, or withdrawing from less profitable markets, while distributors engage in arbitrage. Meanwhile, health providers sometimes protect profits by 'upselling' patients to medicines not covered by insurers. Cost-cutting can undermine quality assurance, leading to substandard or degraded medicines. Other responses contribute to shortages, irrational demand and high prices. All of these provide market opportunities for producers of falsified products; they also push consumers outside of the regular supply chain, providing falsifiers with easy access to customers. The analytic framework capturing these interactions explained cases in most high and middle-income settings; additional factors operate in the poorest countries.

**Conclusions:** Most efforts to secure medicine quality currently focus on product regulation. However, our research suggests market mechanisms are key drivers for poor quality medicines, including where political commitments to universal health coverage are under-resourced. We have developed a framework to guide country-specific, system-wide analysis. This can flag risks and pinpoint specific actions to protect medicine quality, and thus health.

## Introduction

Substandard and falsified medical products undermine the prospect of achieving effective universal health coverage (UHC) ^1^. They waste money, fail to cure sick people, and sometimes even kill them. Reviews of *in vitro*, animal, observational and modelling studies conclude that anti-infective medicines that deliver sub-therapeutic doses because of poor formulation or degradation also encourage drug-resistant infections ^2, 3^. A 2017 analysis of 1,500 cases of suspect medicines reported to the World Health Organisation Global Surveillance and Monitoring System for substandard and falsified medical products (the WHO GSMS database) showed that they exist in all regions of the world and affect all classes of medicines. Recently published meta-analyses of prevalence studies suggest that between 10 and 20 percent of anti-infective medicines in low- and middle-income countries might be substandard or falsified ^4– 6^.

The problem is not new. The WHO outlined factors facilitating the manufacture and trade in poor quality medicines and recommended measures to tackle the problem as early as 1999 ^7^. Some 18 years later, in 2017, it published a report whose findings and recommendations were remarkably similar. Both reports grouped factors that facilitate the production and circulation of falsified and substandard medicines into three broad areas:

- Access-related: shortages of affordable, accessible, acceptable, quality-assured medicine;
- Governance-related: weak legislation and sanctions, corruption;
- Limited technical capacity: limitations among regulators, investigators, prosecutors, laboratories etc., as well as among producers and throughout the supply chain.

Other reviews have cited similar factors, and have proposed similar response strategies for prevention, detection and response, underlining, like WHO, the need to strengthen national medicines regulatory agencies ^8^.

This begs the question: if both the drivers of poor-quality medicines and the solutions have been known for two decades, why does the problem persist? We hypothesised that previous approaches correctly diagnose the immediate drivers, but did not sufficiently consider the specific political and economic factors which shape the markets for substandard and for falsified medicines, and which may stand in the way of proposed policy responses.

We thus conducted detailed, qualitative case studies in four middle-income countries which are significant producers and consumers of pharmaceuticals. We aimed to identify specific mechanisms through which political, economic and other systemic factors influence the availability of substandard and falsified medicines and vaccines, and the ways in which they enable or obstruct policies aimed at reducing the production, trade and consumption of those products. On the basis of our findings, we further aimed to develop an analytic framework that can be used at the national level to identify policies and practices that create risks for substandard production or degradation of medical products, or that provide opportunities for market entry of falsified products.

## Methods

Our team consisted of specialists in epidemiology, criminology, medicine marketing and regulation, economics and public policy. We used WHO's 2017 definitions, adopting "substandard" to refer to medical products that do not meet quality standards, and "falsified" to describe those that deliberately misrepresent identity, composition or source, and excluding patent-related considerations ^9^.

Table 1 summarises the steps we took during this two-phase study.

**Table 1.** Summary of study steps.

Phase 1: Deductive approach
Initial literature review: n ≅ 625
Developed initial working theory using critical interpretative synthesis
Developed a draft coding structure for data analysis in NVivo software
Selected countries for case studies, matching countries to key themes identified in initial literature review, working theory, and staff skills
Phase 2: Grounded theory approach
Country-specific review of literature, court reports, institutional & press reports. n ≅ 215
Conducted 88 semi-structured interviews in four countries
Transcribed interviews, translated them into English
Coded interviews in NVivo software
Iteratively identified common patterns and differences in weekly team meetings; developed draft framework
Presented case study results and Market Risk Framework to informal study advisory panel
Integrated feedback; tested & revised framework against 14 cases from the WHO case reporting database
Finalised framework

### Literature review

We began by searching for evidence related to the causes and consequences of substandard and falsified medicines. We searched PubMed, MEDLINE, the WHO Essential Medicines and Health Products Information Portal and Google Scholar using the keywords "counterfeit" (which was, until the 2019 revision, the Medical Subject Heading used for poor quality medicines), "substandard", "falsified" or "poor quality" in combination with "medicin*", "medical products" or "pharmaceutical*". We identified further publications from bibliographies.

We reviewed resulting publications using critical interpretive synthesis—an iterative method designed for analysing heterogeneous qualitative data and developing theory ^10, 11^. Early in the process, we developed a coding structure centred on factors that created a market opportunity for poor-quality medicines, as well as factors that motivate, facilitate, or deter their production or trade. We entered this structure into NVivo software v12 ^12^, using it to code further reading and refining it inductively. It reached near-final form before the country case studies were conducted. We provide the final coding structure along with a log of changes to coding during iterative analysis in the study repository (see *Extended data*; 02_Coding_structure.docx ^13^).

### Choice of countries

We chose to conduct studies in middle-income countries because many, including several with large populations, are now actively pursuing policies designed to achieve or sustain UHC, while at the same time trying to increase domestic capacity for the production of pharmaceuticals. In addition, many are facing a "double burden" of disease, and demand is rising for new treatments for non-communicable diseases, as well as those for neglected and common infectious diseases. Such countries thus provide learning across a wide range of issues; this learning may also be useful to lower income countries that are now considering increasing public financing for health.

In our initial reading, we also identified structural characteristics with potentially significant implications for medicine quality, as described in the Results section below. To the extent possible, we chose to conduct case studies in countries that displayed one of these characteristics. Finally, wishing to search literature and conduct interviews in local languages where possible, we considered the language skills of our research team. 

Once countries had been selected for case studies, we searched national institutional websites as well as the internet for sources relating to substandard and falsified medicines in Chinese, Indonesian, Turkish and Romanian.

### Field research

We developed question guides based on our literature review, with variations for particular sub-studies (see *Extended data*; 11_Interview_topic_guide.docx ^13^). Potential respondents were purposively selected based on their knowledge of the manufacture, regulation, trade, prescription or consumption of medicines; further respondents were suggested by participants. We contacted them electronically and explained the purpose of the study. Consenting respondents (n=88) were provided with further details in writing in their native language, including around procedures to maintain confidentiality, then interviewed face-to-face or by Skype for between 60 and 90 minutes. Consent to record was sought. Where granted (n= 65) consent was repeated on tape. Where denied (n=14) or technically impractical (n=10), consent to take written notes was sought, and written consent for interview was obtained. Most interviews were conducted in the respondent's native language; staff of international organisations were interviewed in English.

### Data processing, analysis and testing

Recorded interviews were transcribed and translated into English in full or in part by the interviewer. Where no recording was possible, notes were typed in English. Researchers coded their own interviews in a shared NVivo project; the principal investigator (EP) coded a subset of interviews in parallel—differences in coding were discussed in weekly team meetings to develop shared understanding around key concepts. More details of participant recruitment, interviews and data handling, reported following COREQ guidelines ^14^, is provided in the study repository (see *Reporting guidelines*; 03_COREQ_medicine_quality_study_info_form.docx ^13^).

We used a grounded theory approach in developing a coherent framework based on the similarities between country case studies ^15^. A draft analytic framework was developed and presented to an informal study advisory group in April 2018 (see *Acknowledgements*). It was revised following input from the group.

We then collaborated with colleagues at WHO, asking them to identify cases they considered illustrative of the breadth of reports of substandard and falsified medicines logged in the GSMS database. The selected cases included reports from the Democratic Republic of Congo (×2), Nicaragua, Niger, Pakistan, the United Kingdom, the United States and Venezuela, as well as cross-border and regional cases involving China, France, India, Paraguay, the Middle East and West Africa (×3). We mapped these cases on to the market risk framework, to ascertain the extent to which the factors identified in the framework explained the dynamics of each case.

### Ethical approval

The Daily Board of the Medical Ethics Committee of Erasmus University reviewed the study plan and questionnaires and approved the protocol with number MEC-2018-016. National guidelines in all of the concerned countries specify that local ethical approval must be sought for invasive, but not non-invasive research. We discussed the study with officials in the Ministry of Health and/or medicine regulatory authorities (at the provincial level in China and at the national level in other countries) before the start of data collection to ensure that they were fully aware of the aims of our work.

Study funders were consulted over final study design; funders had no role in analysis, manuscript development or decision to submit for publication.

## Results

These results bring findings derived from our review of literature and documentation together with data collected in semi-structured interviews. Where no specific reference is given, the finding comes from interview data.

Altogether, we reviewed some 840 papers and documents relating to medicine quality and product falsification in seven languages, and interviewed 88 people. We provide a full downloadable bibliography of all documents reviewed.in the study repository (see *Extended data*; 04_Bibliography.ris ^13^).

## Global overview from literature: contributors to poor quality medicines

Many of the academic research papers we found reported surveys of the prevalence of poor-quality medicines in low income countries ^6^. The academic literature also provided many commentaries that summarised existing evidence. Some stressed the importance of market factors ^16^, and several called for greater political commitment to addressing the threat posed by poor quality medicines ^17– 19^. However, few of these provide any detailed analysis of the reasons why that commitment is currently lacking.

Books and institutional reports were more likely to provide information about factors facilitating the circulation of poor-quality medicines, as described in the *Introduction* ^4, 5, 7, 8, 20– 25^. These documents generally agree on the circumstances commonly associated with poor quality medicines: shortages of affordable, quality-assured medicine; weak legislation and sanctions; under-resourced regulators with poor technical capacity; complex supply chains; and corruption. We used these areas of agreement to inform our coding structure and interview guides.

However, we found no global analyses which described how these factors relate to one another, or to the political and economic landscapes in which they are rooted.

Much of the remainder of the literature fell into two categories: studies of the medicine markets in a particular country or trading area, and investigations of cases in which falsified or substandard medicines reached markets ^26– 31^. These provided additional insights about factors likely to affect medicine quality, especially in middle-income markets, and contributed to our choice of country case studies. Salient factors increasing risk included market structures that facilitate arbitrage or parallel trade (to which Romania is subject), very rapid scale-up of health service provision (all study countries), decentralised or otherwise complex government and regulatory structures (Indonesia, China). Protective factors included effective single-payer systems and strong information systems, both present in Turkey.

## Country case studies

Table 2 provides key characteristics of the countries selected for case studies.

**Table 2.** Characteristics of countries studied.

Variable	China	Indonesia	Turkey	Romania
Population 2016 (million)	1,379	261	80	20
World Bank Income classification	Upper middle	Lower middle	Upper middle	Upper middle
Health spending per capita, (US$ PPP 2015)	779	383	1029	1128
Annualised growth in health spending, 1995–2015 (%)	10.1	5.6	3.1	5.9
public or insured % of health spending (2015)	67	52	83	79
Generics as % of domestic drug consumption, by volume	80	70	56	60
Value of domestic pharma production, 2016 (US$ billion)	249	3 (2014)	17 (2015)	3
Health financing model (with % covered, 2018)	Social health insurance	Single payer insurance (74%)	Single payer insurance (98%)	Single payer insurance (74%)
Focus of country sub-study	Production of API	Scale up of national insurance	Track and trace	National and regional regulation

API, active pharmaceutical ingredient. Sources: World Bank and International Health Metrics and Evaluation databases (available through 33, 34)

The particular focus of each country case study influenced the choice of informants approached for interview. China is the largest producer and exporter of active pharmaceutical ingredients (API) in the world; it also has a complex decentralised regulatory structure. We chose to focus on the interaction between international, national and state-level factors influencing quality assurance of Chinese API in Zhejiang province, the country's largest producer and exporter of active ingredients and home to four of the world's top ten producers, according to trade sources ^32^. We thus spoke principally to manufacturers and regulators in Zhejiang.

While health services in Indonesia were decentralised in 2001, the central government committed in 2014 to including all citizens in a single-payer health insurance scheme. Though three-quarters of the population is now covered, the scheme has been in deficit since its inception. We focused our case study on the interaction between the radical changes to health financing and the quality of medicines, speaking to stakeholders across the system.

Turkey undertook a similar transformation in health financing a decade earlier, and has more recently invested in widely praised medicine tracking technology. The Turkish case study paid particular attention to the genesis of this technology, and the factors that facilitated or threatened its implementation; many of the interviewees were directly involved with or impacted by these efforts.

Romania is one of the newest and poorest members of the European Union; out-of-pocket spending on health is close to 40% higher than the EU average ^33^. Successive governments have promised to reduce medicine prices, but must do this within the EU's single market, while Romanian pharmaceutical companies must comply with EU standards. The Romanian case study included interviewees from across the supply chain, and highlighted the interaction between national and regional goals, regulations and markets.

Table 3 shows the number of interviewees in each country case study.

**Table 3.** Number of people interviewed, by country and respondent type.

Interview subject	China	Indonesia	Turkey	Romania	Total
Manufacturers/Pharma industry groups	5	4	2	3	14
Brokers or distributors	5	2	1	4	12
Health care practitioners	-	8	1	5	14
Ministry of Health	-	4	3	2	9
Medicine regulator	7	2	*	1	10
National insurer	-	1	2	1	4
Technical agencies for pharmaceutical policy	2	2	6	3	13
Academic	-	2	1	-	3
Patient, media, civil society	-	6	-	3	9
Total	19	31	16	22	88

The particularities of each country focus will be reported separately. The remainder of our results present the common dynamics that we found across these different country case studies, and the analytic framework that arises from them.

## Political promises and policies affecting the market for medicines

We found that political promises, and the policies intended to deliver on them, fundamentally shaped the market for medicines in all study countries, with more uniformity on the demand side of the market than on the supply side. Because the Chinese study focused on the API market, we do not include specific information on the demand side for formulated medicines in China.

### Demand for medicines

In all of the study countries, governments have promised to provide virtually all citizens with affordable access to health care. Scaling up service provision to deliver on this promise clearly increases the size of the market for medical products.

The health financing mechanisms that aim to deliver UHC differ in Indonesia, Turkey and Romania, but all include high degrees of public subsidy. This, combined with rising demand, puts pressure on public budgets, and makes cost containment a priority. Procurement policies have thus increasingly focused on bringing down the price of medicines. Other health system measures have reinforced these efforts, as shown in Table 4.

**Table 4.** Policies aimed at containing the cost of medical products.

Measures	Indonesia	Romania	Turkey
**Procurement measures**			
National formulary	Yes	Yes	Yes
Internal reference pricing	Yes	Limited	Yes
External reference pricing	Yes		Yes
Other pricing policies	Yes	Yes	Yes
Global budgeting	No	No	2010-2012
Restriction to INN generics	Yes	Yes	No
Public auctions/price transparency	Yes	Yes	Yes
**Other health system measures**			
Clinical guidelines	Limited	Yes	Yes
Health technology assessment	Limited	Yes	Yes

INN, International non-proprietary name. Sources: Interviews and 7, 41, 42.

Some of these measures are incomplete or inconsistently implemented. For example, infrequent updating of prices in response to changes in reference prices or exchange rates disrupted markets in Romania and Turkey. In Indonesia, inclusion on the national formulary precedes cost-effectiveness analysis (which is only performed for limited classes of drugs, such as oncology). This reduces the state's bargaining power in auctions.

However, taken together, cost-containment measures have effectively reduced the price paid for many medicines in the study countries ^35– 39^. 

### Supply of medicines

China's government includes pharmaceutical industry growth in its national development plan, “ *Made in China 2025*” ^40^. In all countries, however, we found many, more general political promises that have implications for the supply of medicines. We group them under the category of "national prosperity"; they encompass areas as diverse as economic wellbeing, environmental health and promotion of religious interests.

Table 5 details some of the policies we encountered, with their real or potential effect on the supply of medicines. Many of these effects are unintended, but they have very real consequences for companies seeking to supply the medicines market.

**Table 5.** Policies affecting medicine supply in study countries, and their effects.

Political promise	Country	Policy	Effect
Economic growth	Romania	EU membership	Pharma producers subject to EU standards; single market
Domestic jobs	Indonesia	Minimum 40% domestic components	Threatens Indonesian pharma, which imports >90% API
Healthy environment	China	Reduce polluting industry	Closure of major API producers
Protect religious interests	Indonesia	Halal law, requires halal certification of all medicines	Potential withdrawal of imported products
Fiscal responsibility	Romania, Indonesia	Taxation, incl. clawback tax	Increases tax burden on pharmaceutical firms

Sources: Interviews, and 44– 47

While membership of the EU promised prosperity for Romania, for example, compliance with European good manufacturing practice (GMP) regulations pushed the price of domestic medicines up by over 30%, and led to the closure of some companies, according to reports in the financial press ^43^.

Similarly, in China, thousands of factories manufacturing chemicals for the medicine market were closed down as a consequence of policies designed to improve the physical environment. This increased the price of some products, and created severe shortages for others.

*“Our company’s sales revenue dropped 4% [in 2017]. In monetary value, we lost 100 million RMB on sales. The most important reason was that many chemical manufacturers were closed down. Now the situation is: we have clients, for example those from India request certain products, but these products are no longer manufactured. So, it’s very frustrating that we have business opportunities, but no products to sell.”*

                                                   Chemical/pharmaceutical trading company employee, China

The policies affecting the supply side have wide-ranging effects, but the majority that we encountered tend to increase the cost of producing medicines or of importing them in to national markets.

### Combined market effect

**Figure 1.**
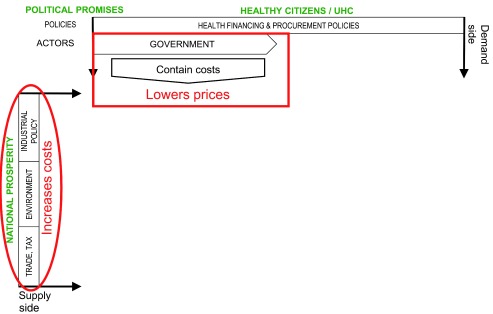
The effect of political promises and policies on medicine markets.

Most markets are the product of supply and demand. Figure 1 shows schematically how political promises and the policies that operationalize them shape the market for medical products in the countries we studied. The combined effect of lower prices and higher production costs is to squeeze profit margins for producers and distributors of medicines. And yet most of those companies—domestic and multi-national, innovator and generic, and including the state-owned enterprises in the countries we studied—are profit-oriented and will act to protect their profits.

We thus turn our attention to the actions they take, which we cluster into three areas: cost-cutting, avoidance of loss-making markets and products, and promotion of high-margin products. For each area we describe the action, the results of that action, the steps that are taken to mitigate the risks by medicine regulators and others, and the limitations of mitigating action.

## Market response 1: Industry protects margins by cutting costs

Where procurement or other policies make it hard to raise prices, producers and distributors of medicines try to protect margins by cutting costs. They may increase efficiency, seek economies of scale or increase mechanisation. They may also seek cheaper suppliers or skimp on quality assurance procedures.

*Q: Is there an effect, where, because of the low offering prices, the components of medicines are compromised?*
*A: Yes, definitely. Starting with the raw materials. That's the first thing, manufacturers are going to look for the very cheapest API, they're going to look for a cheaper supplier. Next is the way they make the medicines available. For example, they might have started with blister packs, but they'll change those to strips, something cheaper. Basically, they're looking for ways to make more profit.*

                                                   Pharmaceutical manufacturer, Indonesia

An API producer in China confirmed the close link between price and product quality, reporting that controlling a particular impurity at 0.1% or less could increase the price of a chemical by 20% or more. Prices of Chinese API vary by destination market; importers in the United States pay approximately three times as much per kilo on aggregate as do importers in Africa ^48^.

### Risk to product quality: substandard production or degradation

Reducing the quantity of active ingredient to save money will lead directly to a substandard product; buying cheaper API is likely to do the same. Lower-cost excipients may alter the way a pill dissolves or otherwise reduce its bioavailability, and thus its efficacy, and may also increase the risks of degradation.

Choosing cheaper packaging or under-investing in distribution mechanisms, including adequate temperature control, increases the likelihood of degradation ^49^. Meanwhile, cutting corners on quality assurance creates risks of mistakes during the production process; this can cost hundreds of lives, for example when breaches of standard operating procedures lead to chemicals being switched inadvertently ^50^.

**Figure 2.**
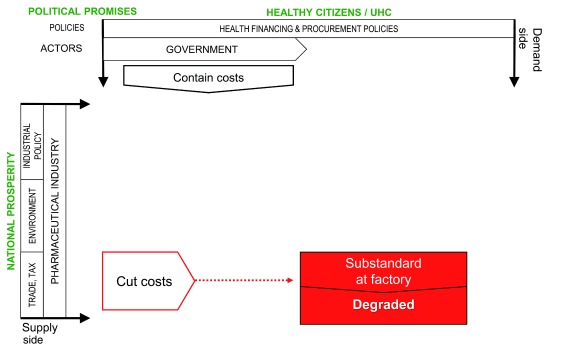
Cost-cutting in defence of profit creates risk for substandard medicines.

These effects are represented schematically in Figure 2.

### Mitigating actions: inspection, surveillance, prequalification

Cost-cutting should not affect product quality as long as producers observe GMP and distributors adhere to good distribution practice (GDP). In most countries (including all study countries) the medicine regulator periodically inspects factories and supply chains, and issues GMP and GDP certificates.

However, in part because of limited resources, this process is far from water-tight. Political pressure to protect local industry also sometimes obstructs nationally-mandated inspections:

*“Sometimes regulation can be difficult. For example, we are not encouraged to impose tough regulation on some enterprises which are highly valued by local governments. Local governments will step in and protect those enterprises.”*

                                                   Medicine regulator, China

The result of inspections by regulators are not always taken into account by purchasers. In Indonesia, for example, a valid market authorisation is the only indicator of product quality required in public procurement ^41^. However, market authorisations remain valid for five years, and are not routinely revoked if GMP certification is withdrawn following an inspection, allowing continued procurement of potentially substandard products.

National regulators are not mandated to inspect production or distribution of products for export (although the EU requires exporting country regulators to provide GMP certifications for the API it imports). The onus for quality assurance thus lies with the importer's regulator, but few countries have the resources to send inspectors to plants in countries from which they buy products, while pre-import GDP inspection is largely absent ^51^.

A Chinese manufacturer explained how this may lead to a "two-track" system for product quality:

*“If the requirement is 0.1% [maximum impurity], a good manufacturer would refine up to 0.07-0.08%.... But some manufacturers would want to save cost and just reach the borderline point. It happens that test result from our clients shows 1.1% and they can’t accept the product. …What happens next would depend on the status and bargaining power of both sides. If we supply [a top-10 multinational pharmaceutical firm], no doubt the product will be returned and we will have to make new ones for them. If we supply middle-end customers whose downstream markets are not well regulated, for example the African market – we are not discriminating Africa, but it’s true their state regulations are not so strict – in that case, we may be able to persuade the client to use the product, by offering for example 5% further discount.”*

                                                   API manufacturer, China

We also found evidence that even the most stringent regulatory authorities take the needs of their domestic industry into account when considering enforcement of GMP, including in other countries. A Chinese manufacturer described the outcome of an inspection of their factory by the United States Food and Drug Administration:

*"The USFDA [issued an import prohibition] for all our products at first. But for a few of our products, [we] are the unique producer, and the US often faces shortages of these products, so FDA very soon released the prohibition on these products."*

                                                   API manufacturer, China

Importing countries unable to inspect factories in exporting countries may choose to buy products that are quality-assured at source under a WHO-implemented prequalification system ^52^. This system currently only covers medicines or vaccines for a few infectious diseases, and some reproductive health products.

Where GMP/GDP inspection at/from source is not an option, regulators may attempt to mitigate the risk of substandard products reaching patients through post-market surveillance. However, we found that in low and middle-income countries most post-market surveillance is performed using screening tools capable of spotting falsified medicines and incorrect API, but inappropriate for identifying poorly formulated or degraded products ^53^.

Where substandard products are identified, full track-and-trace systems such as that in use in Turkey greatly facilitates rapid recall, minimising public health damage.

## Market response 2: Industry protects margins by avoiding unprofitable products or markets

A second response to low margins is to simply avoid products or markets that do not contribute to a company's profits. In the three years after Romania effectively capped prices at the EU minimum while charging producers a claw-back tax, manufacturers withdrew around 2,000 of 6,200 products ^39^. Many of these were lower-priced generic medicines.

*"Authorities [explaining supply interruptions] say 'No, no, you've had production problems'. But the truth is that it's very simple to explain the decision not to produce a drug as long as it brings you losses."*

                                                   Technical agency advisor, Romania

While domestic companies stop manufacturing loss-making products, multi-national firms, whose profit calculus is global, have the option of simply avoiding loss-making markets. They may do this for a single product, by refusing to register it in country, or they may withdraw from a country entirely. One multinational firm shut down their generic production lines in Indonesia after the government introduced new procurement rules to contain medicine costs, citing commercial pressures ^54^.

Multinational manufacturers also stay away from low-priced markets to avoid being benchmarked by countries that use external reference pricing to establish domestic prices.

*"Innovative medicines do not come…because they know the Turkish market will pull down prices [in other markets], unfortunately. [Manufacturers] do not want to get themselves into something like that."*

                                                   Academic pharmaceutical market analyst, Turkey

Finally, manufacturers restrict distribution of expensive products to countries with lower prices for fear that profit-seeking brokers will buy them up and resell them in higher-priced markets, thus eroding the manufacturers' profits. Both Turkey and Romania reported this behaviour, although Romania was especially vulnerable because trade regulations allow for the free trade of medicines between EU countries that implement different national medicine pricing policies.

Distributors can reduce costs by distributing only to areas with good infrastructure. In Indonesia, authorities turn a blind eye when distributors protect margins by withholding products from remote islands, where distribution costs are high:

*"Most important for the authorities is that there's no nationwide stock-out. They just let [a shortage] go for remote regions, because they recognise [the government] auction prices are very low."*

                                                   Pharmaceutical manufacturer, Indonesia

### Interim risk: Shortage of clinically indicated medicines, incentive to buy from unregulated suppliers

The result of all of these actions: shortages of quality-assured, clinically indicated medicines.

*"In order to gain political capital, [campaigning politicians] kept saying “we’ll give you the cheapest medicine in Europe.” Wow, how great they are! We’ll vote for them! Without thinking that you won’t have access [to the medicines]. Because no one brings them anymore, because it’s not economically justified anymore, it’s not a business anymore. And those that are economically viable leave the country through parallel export. Romanians end up without medicine in one case or the other."*

                                                   Patient advocate, Romania

Both patients and physicians respond to these shortages by stepping outside of the regulated supply chain to acquire the medicines they need. A Romanian doctor, showing researchers vials of magnesium sulphate with no expiry date, carried across the border by a former patient in response to shortages, said:

*"What do I know about this vial? When it expires? I tell you, I know for how long I have it and if it turns yellow then for sure it’s not good anymore…. What does it do? It saves two lives. Of the mother, and of the baby…. [But] it may be plain water or poison."*

                                                   Doctor, Romania

### Mitigating actions: system-wide planning; market making

Market withdrawal and associated responses are both commercially rational and completely legal; they are responses over which the medicine regulator has very little control. Prevention of shortages of this type requires system-wide planning that accurately forecasts demand, and that incentivises adequate supply, for example by providing guaranteed sales to auction winners.

Strong information systems are a prerequisite for effective planning. Turkey, whose well-resourced medicine regulator operates semi-autonomously under the Ministry of Health, has the most effective information systems of the study countries. These include a Datamatrix system that tracks every transaction involving a medicine in the regulated supply chain, from manufacture to dispensing. The system was implemented in response to widespread reimbursement fraud, estimated at US$ one billion ^55^, that threatened the government's ability to deliver on its political promise of UHC.

The system imposed significant costs on manufacturers, requiring those that wished to sell in the Turkish reimbursed sector to adapt production lines. Even after internal and external benchmarking lowered prices, manufacturers participated in auctions because of the size and consolidation of the market.

*"The customer is king. I pay the money, I determine the conditions. Turkey has such an advantage. [The national insurer] buys more than 80% of the market. They say: “If you are willing to give them under these conditions, then you can give your drugs. Otherwise, I’m sorry, go sell them in another country, don’t sell them to me.”*

                                                   Multinational pharmaceutical manufacturer, Turkey

Reimbursement data matched with data from the track and trace system provides an early warning system for low stocks nationally and locally, allowing for rapid procurement as necessary. Well-managed stocks and an adequate benefits package reduce the likelihood that patients will step outside of the regular supply chain in Turkey.

Indonesia also has a single-payer national health insurance, and has attempted through transparent, single-winner auctions to elicit low bids from manufacturers. However, unlike Turkey, Indonesia's insurer reimburses against diagnostic group; it does not collect data on prescriptions or contribute data to the Ministry of Health's demand planning exercise. In 2017, public sector purchases of paracetamol in Indonesia were just 30% of the amount forecast in tender documents, while for iron folate, they exceeded forecasts by over a quarter ^56^. Bidders must undertake to deliver volumes set in the tenders. However, there is no concomitant obligation on health providers to purchase those volumes, leaving manufacturers with potentially expensive excess capacity and disincentivising participation in auctions.

Romanian regulators guard against shortages resulting from arbitrage by requiring daily reporting of medicine exports. Given recurring shortages, it is unclear whether these data are analysed ^38^.

*Q: "Isn’t there a plan in place to prevent shortages [caused by parallel exports or market withdrawal]?"*

Respondent 1 laughs uncontrollably.

Respondent 2, laughing: *"If there is such a thing, it obviously serves no purpose."*

                                                   National technical agency advisors, Romania

## Market response 3: Industry and health service providers promote profitable products

Besides minimising losses, the pharmaceutical industry acts to maximise profitable sales. In all study countries, producers and regulators alike reported that companies actively lobby to get higher-margin products on to national formularies, as well as to encourage orders for these products from private sector hospitals and others with discretionary buying power.

As countries scale up public financing for health using cost control measures such as reimbursement by diagnostic group, profit margins for private providers of health services also come under pressure. Some seek to maintain income by 'up-selling' patients to medicines that are not covered by national health insurance, for example selling them originator brands rather than giving covered generic alternatives. This reinforces patients' perception that price signals quality, and may contribute to demand for more costly products even when there is no shortage of quality-assured, cost-effective alternatives.

*“In my opinion, if a vaccine is more expensive, then automatically it's going to be better quality, and that’s that.”*

                                                   Mother of infant, Indonesia

### Interim risk: Unmet demand for premium products, incentive to buy from unregulated suppliers

Marketing and prescribing practices that create unmet demand for higher-margin products tend to raise costs to patients. Patients who want, but cannot afford, a premium product that is not covered by insurance may again step out of the regulated supply chain, seeking the desired product over the internet or in informal markets.

Sometimes, health care providers sacrifice due diligence and acquire medicines at a discount, selling them to patients at a premium to maximise profits.

*"Freelance" salespeople go from door to door, including to hospitals, offering drugs sometimes at rather low prices…[The buyer] doesn't think about the improbable price, only about the profit.*

                                                   Pharmaceutical industry association official, Indonesia

### Mitigating actions: control of product mix, licensing of outlets, public awareness campaigns

Most countries try to restrict irrational use of medicines using the policies listed in Table 4. Of these, only market authorisation is generally controlled by the medicine regulator. In theory, market authorisation could be granted only to products that have been deemed cost-effective in health technology assessments ^57^. This in not universally the case in the study countries, so the procedure does not effectively curb irrational demand.

Regulators try to guard against freelance and cut-price sales and other unethical practice throughout the supply chain by certifying pharmacies and other dispensing outlets. Full track and trace systems, such as that implemented by Turkey, prevent the insertion of falsified medicines into the regulated (but not the unregulated) supply chain.

*"There is a “handshake protocol”. When you give it to me, you make a sale-notification, but it is still up in the air. If you do not make that sale-notification, and if the system says when I scan the product: “There is no such thing,”, then I’ll tell you: “I cannot buy this, because I cannot sell this.”*

                                                   Multinational pharmaceutical manufacturer, Turkey

Some countries have launched public awareness campaigns to warn patients of the dangers of buying medicines from unregulated sources. We found little evidence of the effectiveness of these strategies. Rather, the most effective strategy we encountered was that adopted by Turkey, which was to remove the incentive to go outside of the regulated supply chain by ensuring the availability and affordability (in this case through insurance coverage) of quality-assured products.

## Risk to product quality: Market opportunity for medicine falsifiers

**Figure 3.**
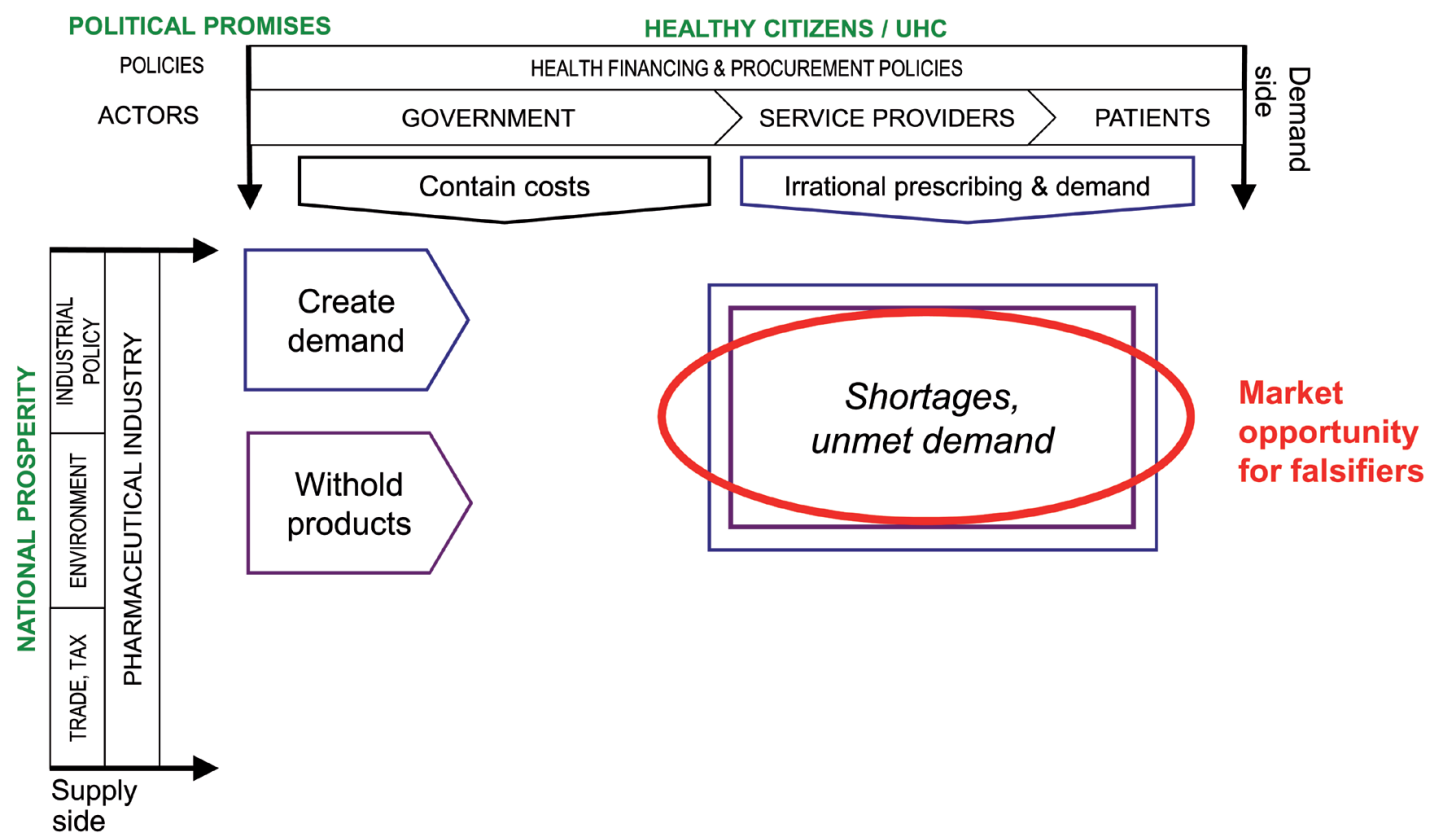
Commercial responses to low margins create market opportunities for medicine falsifiers.

Commercial strategies to protect profit margins combine to create unmet needs and demand, and to push people to satisfy them outside of the regulated supply chain. Together, this creates a market opportunity for criminals who wish to sell falsified products. These dynamics are represented in Figure 3.

We observed these dynamics in both Romania and Indonesia. In Romania, the main driver was shortages of clinically indicated products due to product withdrawal and arbitrage.

*Coming back to your question – why patients buy medicines from the internet – you have to know that the majority of them do this in full knowledge, because the state does not provide them with a treatment in time. They are desperate and take the risk and buy medicines from wherever they can. Desperation pushes them into consciously aggravating their own situation, because is it known that many of these meds are fakes...And they bought them online, and also, from around the corner of the hospital. The saddest thing is that this trade also takes place next to the oncology institute.*

                                                   Patient advocate, Romania

In Indonesia, though quality-assured domestic vaccines were available for free, physicians maximised profits by charging patients for expensive imported vaccines. At a time when there was a supply shortage of imported vaccines, some physicians based in private hospitals bought products at cut price from roving salesmen. In 2016, at least 1,500 children were injected with falsified 'imported vaccines', containing no active ingredients ^58– 61^.

### Mitigating actions: surveillance, criminal investigation

While full track and trace systems can keep criminally produced products out of the regulated supply chain, they do not operate in informal markets.

As long as a market exists, and the likely profit outweighs the likelihood of getting caught, successfully prosecuted, and sentenced to significant loss of liberty or assets, criminals will sell falsified medicines. In many countries, penalties are slight and investigative mandates unclear. Limitations on the Indonesian regulator's authority at the time of the 2016 vaccine scandal facilitated the trade of falsified products in a supposedly regulated supply chain.

*“Regulation-wise, at that time, we were not allowed to inspect pharmaceutical services in hospitals. The parliament pushed us to know more [about the falsified vaccines]. But there was no way we would know about this in the first place. We could not get into those hospitals' systems.”*

                                                   Medicine regulator, Indonesia

Once falsified medicines are in circulation, effective post-market surveillance may interrupt their pathway to patients. Again, however, the reach of regulatory systems in unregulated markets is limited. The high-end products most attractive to falsifiers are also the least likely to be tested, because of the high cost of acquisition and reference standards.

Table 6 summarises the responses to market pressures that we encountered in study countries, and their effects.

**Table 6.** Response to pressure on profits in study countries, and their potential effects on medicine quality.

Goal	Action	Interim risk	Potential risk to product quality	Preventative action by medicine regulator	Preventative action by others
Reduce production costs	Use cheaper API		Higher impurities	Domestic production: GMP inspection Imports: only authorise prequalified products	
	Use cheaper excipients		Higher impurities, less bioavailability		
	Use cheaper packaging		Degradation		
	Use less API		Sub-therapeutic dose		
	Bypass quality assurance		Production errors		
Reduce distribution costs	Under-invest in control of temperature/humidity		Degradation	GDP inspection. (For imports, only possible after arrival)	
	Limit geographical reach	Localised shortages	Market opportunity for falsifiers, including in health facilities		
Avoid loss- making products	Cease production	Shortages	Market opportunity for falsifiers, including in health facilities		Fair pricing
Avoid loss- making markets	Withdraw from market				
	Fail to register in market				
	Limit distribution				
Seek profitable markets	Arbitrage	Shortages in source market	Market opportunity for falsifiers including in health facilities		Fair pricing
	Aggressive market entry	Irrational demand for high priced product	Market opportunity for falsifiers, especially targeting patients	Consider HTA in granting market authorisation	National formulary, HTA
	Aggressive marketing				Clinical guidelines
Maximize profits from patients	Up-sell to off-plan products				Comprehensive insurance coverage

API, active pharmaceutical ingredient; GMP, good manufacturing practice; GDP, good distribution practice; HTA, health technology assessment.

## Market risk framework for substandard and falsified medical products

**Figure 4.**
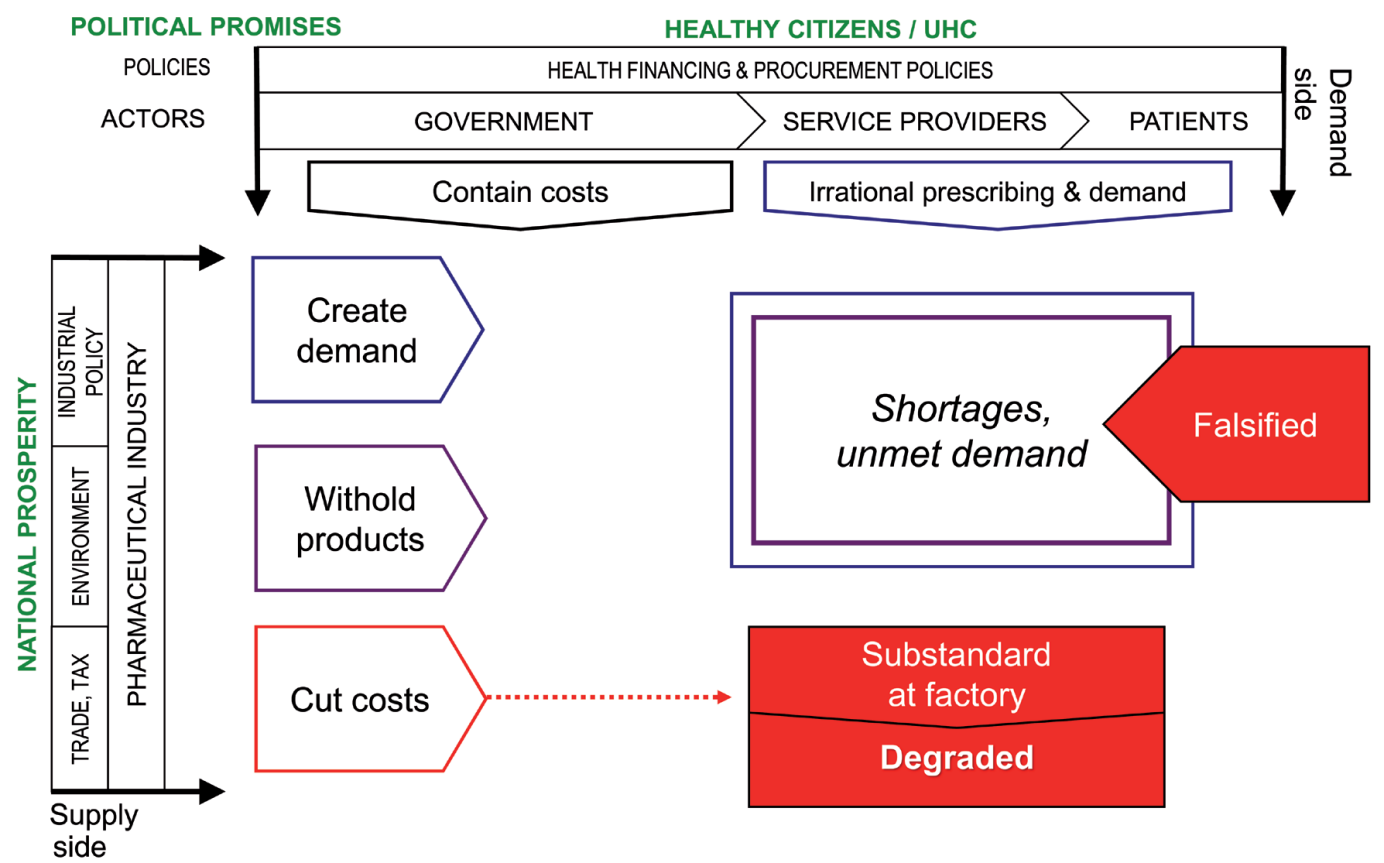
Identifying substandard and falsified medical products: a market risk framework.

Figure 4 brings all the elements discussed together into a single framework, which we dub the "Market risk framework" for assessing the likelihood that substandard and falsified medicines will reach a nation's patients.

**Figure 5.**
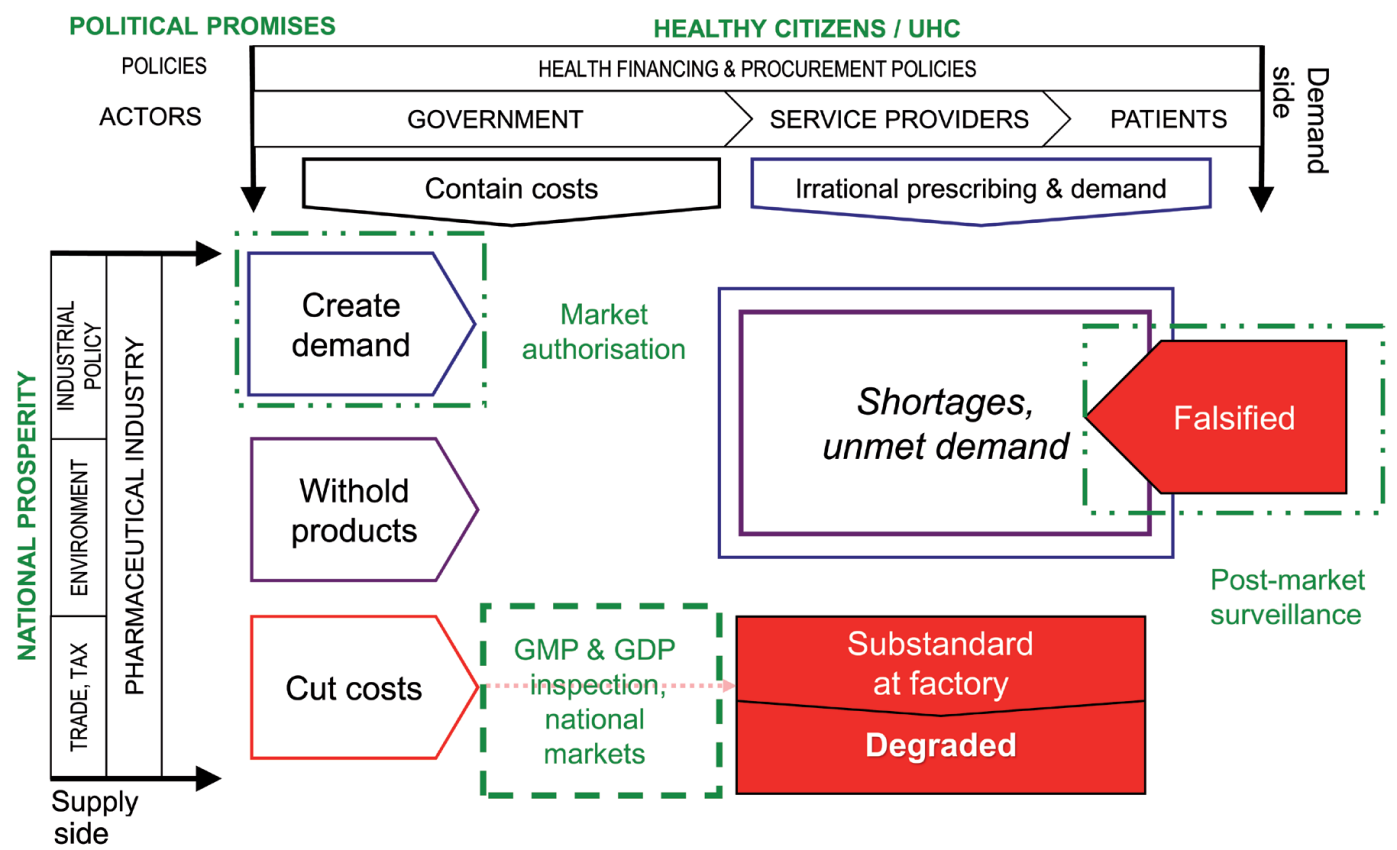
Actions by national medicine regulators mitigating market risk.

Figure 5, meanwhile, adds information which illustrates the points at which action by national medicine regulators may mitigate risk.

A narrated video walking through the framework is available in the study repository (see *Extended data*; 01_Pisani_et_al_medicine_quality_market_risk_framework.mov) and on Vimeo.

In the study repository, we provide versions of the framework describing the specific factors that create risks for substandard and falsified medicines in our study countries, fully annotated with data from the studies (see *Extended data*; 05_China, 06_Indonesia, 07_Romania and 08_Turkey ^13^).

## Testing the framework

We tested the framework against cases selected by colleagues at WHO from the GSMS database, as described in the methods section.

To illustrate the results from countries at different income levels, we provide brief annotated versions of the framework for GSMS cases with data in the public domain from the United States, Pakistan, and Democratic Republic of Congo. These can be found in the study repository (see *Extended data*; 09_WHO_database_case_studies.docx ^13^).

Overall, we found that the framework developed in middle-income countries with aspirations to achieve UHC explained most of the factors driving the falsification of medicines in countries of all income levels, as well as the production and import of substandard medicines in lower income countries. However, some additional factors emerged in low income countries:

- Substandard production is sometimes simply the result of limited technical capacity among producers;
- Where most spending on medicine is out of pocket, people buy in informal markets for convenience and affordability, even when there are no shortages;
- In heavily aid-dependent countries, procurement practices of donors can undermine national efforts to build sustainable markets and systems.

In addition, the framework, which focuses on national drivers and deterrents, does not adequately capture the potential for cross-border exchange of information in guiding GMP inspection and post-market surveillance.

## Discussion

Substandard and falsified medicines have increasingly been identified as a neglected challenge to global health ^62, 63^. Efforts have been made to describe the causes of poor-quality medicines, and to identify appropriate responses to the threat they pose ^4, 7, 8, 22^ However, none has provided a comprehensive, evidence-based framework which simultaneously:

- Elucidates the system-wide policies that incentivise and facilitate the production, trade and consumption of poor quality medical products;
- Differentiates those that drive substandard production and degradation from those that create opportunities for falsification;
- Maps specific regulatory responses on to specific pathways, highlighting the likely effects of different investments and policy choices.

By comparing detailed case studies in four countries, and testing the results against recorded cases in other countries, our work has provided a framework which disentangles the many factors previously grouped under broad headings such as "limited access to quality medicines", "poor governance" and "restricted technical capacity". We provide granularity to help guide national plans to prevent, detect and respond to substandard and falsified medical products.

The study shares the limitations common to qualitative research regarding subjects involving illegal or unethical behaviour. While we worked hard to include a wide range of participants and to stress that data would be anonymised, some potential respondents declined interview while others, particularly from government institutions, gave normative responses. However, we were able to capture a wide range of opinions and experience, including detailed descriptions of unethical practices, giving us confidence that our results are sufficiently comprehensive.

The four middle-income countries studied shared characteristics that are not reflective of all nations; when we tested the framework against cases reported in other countries, we identified refinements which may be necessary in adapting the framework for lower income settings.

Our results did, however, prove robust across a wide range of settings. They suggest that when the incentives driving demand for affordable, cost-effective medical products are aligned with the rewards for producing and distributing those products at assured quality, substandard medicines will be rare, and there will be little opportunity to profit from the sale of falsified products. However, it is currently hard to align production incentives with rewards, because the forces shaping demand are largely determined by national governments aspiring to maximise access to affordable care, while suppliers are motivated by profit, often calculated globally.

Much of the current discourse about medicine quality focuses on strengthening the capacity of national regulatory agencies to oversee the production or import of medicine products, and their distribution. Certainly, well-resourced national medicine regulators are critical in assuring product quality. However, our analysis shows that many policies far beyond the reach of the medicine regulator contribute to shaping the market for medicines, sometimes incentivising the production or import and sale of substandard or falsified products.

We find that unless quality is explicitly included in pricing and procurement policies, downward price pressures can actively incentivise the production of substandard medicines and facilitate degradation. No country can hope to achieve sustainable, effective UHC without quality-assured generic products, yet low-profit-margin generic products are especially vulnerable to corner-cutting in response to price pressures.

A key safeguard is thus to ensure that prices cover quality production and distribution, as well as fair profit. This is an unpalatable message. "Fair profit" is hard to determine, since manufacturers rarely disclose real costs ^64, 65^. Further, profit is often conflated with profiteering in discussions of pharmaceutical pricing, while the fear that innovator producers hope to undermine public confidence in generic products discourages discourse around quality assurance for low-cost products. Meanwhile, large exporters of generic products often respond to calls for more quality assurance by accusing more expensive producers of covert protectionism ^66– 69^.

A further safeguard against substandard production is close regulation of manufacturing practice. We find evidence, however, that a political commitment to the promotion of domestic manufacturing may put pressure on regulators to lower standards for GMP or product quality inspection. This is worrisome in a climate of increasing economic nationalism in many middle-income countries, including those needing to find new sources for medicines previously supplied by the Global Fund for AIDS, TB and Malaria ^70^.

The current global system concentrates the burden for quality-assurance on medicine regulators in over 190 consuming countries, rather than in a handful of major exporting nations. Adequate quality assurance of products made for export would increase efficiency in a globalised pharmaceutical market. This is a political rather than a technical challenge, but models exist in other sectors, such as aviation ^71^. More cost-effective, streamlined regulation could eat into fees that national regulators now earn for often duplicative authorisation procedures, but it would cut costs for quality manufacturers, reducing the risk of product withdrawal and shortages.

Product shortages, as well as the quest for affordability or profit, can push patients and health care providers out of the regulated supply chain, creating opportunities for falsifiers. As the Turkish case study demonstrates, the existence of a consolidated market in which both patients and producers are relatively well protected against excessive price pressures is an effective protection against this risk. Market-based solutions such as this appear more effective than attempts to tackle falsification primarily through product regulation ^16^.

## Conclusion

As The Lancet Commission on Essential Medicines observed, UHC cannot be achieved with poor quality pharmaceuticals ^1^. We thus conclude that organisations and governments aiming to ensure UHC must consider the potential impacts of procurement rules as well as industrial, environmental and trade policies on the quality of medicines, and support and implement systemic approaches that provide fair reward for the production and distribution of quality-assured, cost-effective therapies.

There is considerable scope to validate the market risk framework in different country settings, and to use it in combination with well-designed quantitative surveys to better understand the scale of the problem and to address the locally-specific drivers of vulnerabilities to poor quality medical products at the systemic as well as the national level ^72^. Distinguishing between risk factors for substandard medications and falsified products can also guide choices about post market surveillance equipment ^53^.

The framework also functions as diagnostic tool to identify market and regulatory failures that have incentivised or facilitated a specific case of falsification/substandard production. We encourage its adaptation to local circumstances, and its use in ensuring that the call for greater "access to medicines" reliably means "access to medicines that work".

## Data availability

### Underlying data.

The interviews that underlie this study discuss sensitive and at times illegal behaviours. During our informed consent procedure, we assured participants of anonymity. We are unable to comply with that commitment if we make the recordings of the interviews available. We believe there is an unacceptably high risk of disclosure in sharing full transcripts, and believe fully redacted manuscripts may be of limited value.

Our detailed coding structure is published in the study repository. Researchers from universities and accredited public-interest research institutions are welcome to request specific coding queries. Please contact the Medical Ethics Committee of Erasmus University ( metc@erasmusmc.nl), giving your name, institution, and the purpose for which you will use the data, and citing study number MEC-2018-016. Please copy your request to the Erasmus Data Service Centre ( edsc@eur.nl) and to the corresponding author of this paper. If the ethics committee raises no objection, the research team will run the query as requested, redact the results only to the extent necessary to ensure anonymity, and pass the results on to fellow researchers, for the stated use only.

### Extended data

Harvard Dataverse: Supporting data for: Identifying market risk for substandard and falsified medicines: an analytic framework based on qualitative research in China, Indonesia, Turkey and Romania. https://doi.org/10.7910/DVN/0GGI9D ^13^.

This project contains the following extended data:

- 01_Pisani_et_al_Medicine_quality_market_risk_framework.mov
- 02_Coding_structure.docx
- 04_Bibilography.ris
- 05_China_case_study.docx
- 06_Indonesia_case_study.docx
- 07_Romania_case_study.docx
- 08_Turkey_case_study.docx
- 09_WHO_database_case_studies.docx
- 10_Medicine_quality_in_Indonesia.mov
- 11_Interview_topic_guide.docx

### Reporting guidelines.

Harvard Dataverse: COREQ checklist for article “Identifying market risk for substandard and falsified medicines: an analytic framework based on qualitative research in China, Indonesia, Turkey and Romania”. https://doi.org/10.7910/DVN/0GGI9D ^13^.
