# Supplementary material for: Identifying market risk for substandard and falsified medicines: an analytic framework based on qualitative research in China, Indonesia, Turkey and Romania
Source: Wellcome Open Res. 2019 Apr 16;4:70. [Version 1] doi: 10.12688/wellcomeopenres.15236.1 (PMC6518437; doi:10.12688/wellcomeopenres.15236.1)
Supplement: Peer review history

# Peer review: Reviewer response for version 1

**DOI:** 10.21956/wellcomeopenres.16629.r35338

**Published:** 2019-06-05

**Authors:** Prashant Yadav

**Affiliations:** 1 Department of Global Health and Social Medicine, Harvard Medical School, Boston, MA, USA

**Summary**

 This paper attempts to investigate political and economic factors which drive the demand and supply of sub-standard and falsified (SF) medicines. Using a grounded theory approach, the authors try to develop a market risk framework for SF medicines. The main issue addressed by this paper is of significant policy relevance and impact. It is the first paper (to the best of my knowledge) which uses a demand/supply and market risk framework to study this problem.

 Below I point out a few areas in the paper where I would have liked to see greater depth in analysis or clearer exposition of what this analysis tells us with a reasonable degree of confidence, and what parts remains less clear.

****

**Comments **

- The key hypothesis is that SF medicines are largely the result of market responses to demand for lower prices/cost containment, industrial policy and environmental control pressures. There may be other underlying root causes for the prevalence of SF. This is where the authors’ use of grounded theory methodology (and the sampling approach used) falls a little short. The manuscript does not provide much detail (there is some discussion on p13) on how they looked for cases/instances that do not fit the main reasons they have identified, what they found, and did they attempt to look at filling gaps in initial data collected to clarify uncertainties. This would have allowed them to add more depth/density and allow a reader to fully understand which parts of their grounded theory to have stronger confidence in, and which parts to have lesser confidence in. They delve into this a little bit in the discussion section, but it would have been better to see a clearer and more elaborate exposition of the negative cases in the main section which discusses the market risk framework.
- All the countries included in the sample have a high degree of health insurance coverage or some form of pooling. To what extent would the findings of this study apply to out of pocket markets without any price controls? Posing this question would help bring out the external validity scope of this paper more clearly. It will also more clearly highlight other factors that lead to SF.
- It will be useful for the authors to look at Boswell-Dean (2019) ^1^ which shows that price controls in India may have led to higher quality in the out-of-pocket medicines market.
- The drivers for sub-standard and falsified medicines are often very different and the framework developed by the authors and Figures 3, 4 and 5 clearly demonstrate that. It may be useful to categorize them more explicitly in Table 6.
- Shortages (due to different reasons) are identified as the primary driver for falsified medicines. While this may be true for the 4 middle income countries studied, in many other markets, falsified medicines are supplied to out-of-pocket paying patients even when the “genuine” product is in abundant supply (although is priced higher). This sometimes makes the distinction between the underlying reasons for sub-standard and falsified a little murky. Also, see Bate *et al.* (2011) ^2^ for some discussion regarding this.
- While unclear if it applies widely due to the nature of the study sample, it is worth exploring if a possible market response would be to differentiate drug quality according to the consumption sub-market as in Bate *et al.* (2016) ^3^.
- While I understand purposive sampling based on market characteristics, using API market in one country (China) and finished products in the other countries is not a very good comparison strategy for building grounded theory. Cost containment from political pressures only directly effects finished product producers. The effect of cost containment on finished product translates into a much lower price effect on APIs (depending on the type of medicine only a small fraction of the cost is API cost). Also, there is limited evidence that finished product producers pass-on their price decrease pressures to API suppliers. Economics of API manufacturing is also much more volume and fixed cost dependent so its better to make larger quantities and supply them at marginal cost to other “non-consumption” markets.

I have read this submission. I believe that I have an appropriate level of expertise to confirm that it is of an acceptable scientific standard.

## References

[1] Boswell DeanE: Who Benefits from Pharmaceutical Price Controls? Evidence from India – CGD Working Paper 509. *Center for Global Development*.2019; Reference source

[2] BateRJinGMathurA: Does Price Reveal Poor-Quality Drugs? Evidence from 17 Countries.2011; 10.3386/w16854 10.3386/w16854 21917346

[3] BateRJinGMathurAAttaranA: Poor Quality Drugs and Global Trade: A Pilot Study.2014; 10.3386/w20469 10.3386/w20469

# Peer review: Reviewer response for version 1

**DOI:** 10.21956/wellcomeopenres.16629.r35340

**Published:** 2019-05-14

**Authors:** Helen Lambert

**Affiliations:** 1 Population Health Sciences, Bristol Medical School, University of Bristol, Bristol, UK

This is an impressive contribution to a particularly difficult-to-research issue in global health. While the problem of substandard and falsified medicines and its potentially adverse health consequences is well recognised, research has tended to focus on documenting the scale of the problem and enumerating the contributory factors. This paper uses empirical case studies and review of secondary literature to develop a 'market risk' analytic framework for use in mapping the political and economic system dimensions of the problem in specific country settings. The framework is built on a literature review followed by comparative analysis of qualitative interviews with relevant informants and stakeholders in four diverse middle-income countries.  

 This is a complex issue influenced by a range of interwoven factors, many of which are highly technical or difficult to grasp for non-specialist readers (ranging from Good Manufacturing Practice and regulatory standards to diverse market structures, the effects of differing insurance systems and international trade arrangements). Given this, the authors do a good job in general of highlighting the key dimensions at play without losing the reader in technical detail.  Occasionally a little further explication would be helpful, for example in elucidating the role of arbitrage or the practice of auctions through which (it appears) governments select a supplier for a required pharmaceutical product. 

 The study design is generally sound and the use of semi-structured interviews is particularly appropriate for gathering material on sensitive topics such as this. The authors could perhaps have made fuller use of the substantial review of academic papers and press reports that they conducted by using an established scoping review framework and by providing further details of the critical interpretive synthesis conducted (these results are not included). The development of a coding framework based solely on the secondary literature and its subsequent deployment for coding and analysis of the interview data is somewhat unusual. Analysis of the qualitative case study material could have been enriched by combining the use of this coding scheme with a more inductive approach, to allow development of unexpected themes emerging from the interview data. A fully grounded theory approach would also have included testing the authors' hypothesis against divergent/deviant examples. However, the more structured approach utilised here does serve the authors' purpose of framework development effectively, by focusing on the specific political and economic dimensions that may heighten risk for creating markets for substandard or falsified medicines. Importantly, the authors identify different influences operating for these two types of inferior product. The figures provide clear and useful diagrammatic characterisations of the processes described, such as the effect of cost-cutting on the risk of substandard medicines.  

 The conclusions are generally sound though broader statements could be better evidenced, such as the framing assumption of the paper that downward pressures on prices are exerted by political promises to deliver Universal Health Care. Only the implementation of such promises through legislation, regulation or other government actions to reduce costs of medicines rather than the 'promises' themselves are really relevant to this assertion; and since many governments are focusing efforts toward UHC primarily on expanding health insurance coverage, some further discussion of the potential implications of different health insurance arrangements on risks relating to medicine quality would have been helpful. 

 Nonetheless, overall this is a sophisticated and impressive analysis that provides an original conceptualisation that helps to clarify a rather murky field. It provides a potentially useful framework for further country-specific analyses to disaggregate the various contributory factors that may influence susceptibility for low quality and falsified medicines to appear in the market. Moreover, it rightly shifts focus from product regulation, where efforts have mostly concentrated to date, to causal market mechanisms; and it usefully pinpoints the need to differentiate the economic forces likely to result in the appearance of substandard medicines from those that increase risk of falsified medicines being manufactured and marketed.          

I have read this submission. I believe that I have an appropriate level of expertise to confirm that it is of an acceptable scientific standard.

# Peer review: Reviewer response for version 1

**DOI:** 10.21956/wellcomeopenres.16629.r35339

**Published:** 2019-05-13

**Authors:** Charles Clift

**Affiliations:** 1 Centre on Global Health Security, Chatham House, London, UK

This is an interesting study of the drivers of substandard and falsified medicines which develops a market risk framework based on four country case studies.

 While the study identifies a number of factors that contribute to the problem, the methodology is not entirely satisfactory. The choice of countries is somewhat eclectic - it is not quite clear why they were chosen over others. The focus of the case studies is different in each case as is the occupational distribution of the interviewees. 

 The paper would have benefited from a more systematic analysis of the health systems and pharmaceutical policies in each of the countries. The details on p.5-6 are quite cursory. This applies also to the case studies themselves in the extended dataset. 

 With regards to the central conclusion that "increasing political commitment to provision of universal health coverage has led to public procurement policies aimed at lowering prices of medical products" I am not sure that it is supported by the data. Public procurement has long sought to minimize prices, often without due regard to medicines quality. UHC, to the extent that it involves greater public funding of health services and a reduction in the number of patients buying their own medicines, could actually improve the prospects for reducing reliance on falsified and substandard medicines. The fault lies in defective procurement policies that do not properly factor in quality criteria and the reluctance of governments to provide adequate financing. Where there is a substantial generic industry, as the report points out, there is also political pressure not to enforce strict regulation. 

 In that context, the report refers to the need to allow "fair profit" to ensure that quality standards are maintained, but how to achieve that raises a lot of issues that are not pursued in any depth. 

 On specific points:

- The comment about the WHO prequalification programme on p.9 rather understates its importance as the reference makes clear. It also covers diagnostics. 
- On p.15 the list of additional factors in low income countries really needs referencing. 

I have read this submission. I believe that I have an appropriate level of expertise to confirm that it is of an acceptable scientific standard.

# Peer review: Reviewer response for version 1

**DOI:** 10.21956/wellcomeopenres.16629.r35341

**Published:** 2019-04-23

**Authors:** Delia Grace

**Affiliations:** 1 International Livestock Research Institute (ILRI), Nairobi, Kenya

**Specific questions**

- The authors have not made the interview data available. However, the explanation for this is fully satisfactory.

**Overall**

- There is great concern but little understanding about the prevalence, drivers and impacts of sub-standard drugs. This paper is timely. It develops a framework, which while not necessarily definitive, is useful. It gives insight into the issue of fake drugs by seeking to understand the drivers for undesired behaviours leading to poor outcomes.

**Introduction**

- Although there are pragmatic reasons for lumping “substandard” and “falsified” drugs and although this is current WHO terminology, combining the two categories can be mis-leading as they have very different drivers and hence different solutions. 
- It is going too far to say that solutions for poor quality medicines in LMICs are known. There is an argument that the solutions proposed, largely drawn from historical experiences in HICs, are inappropriate and unworkable in the context of LMICs.

**Methodology**

- The choice of countries requires more explanation. How many countries fitted the criteria chosen by the researcher?
- The number of participants per country is rather small, but probably adequate for this time of qualitative study.
- Ethical approval for non-invasive research is rather a grey area. They should refer to national guidelines that say local ethical approval is not needed for non-invasive research. Most countries where we work require local ethical approval.

**Results and Discussion**

- It would be useful to have information on the size of the un-regulated drug sector in these countries.
- The results capture very much the “received wisdom”. It would be interesting to have perspectives of some of the “un-approved stakeholders” e.g. the people who buy drugs on line or sellers of illegal products.
- The paper very well documents the un-intended consequences of well-intended goals. They should be more cautious in suggesting preventative actions as it is very likely these preventative actions will have their own set of un-intended consequences.
- The application to low income countries was perfunctory and a more in-depth analysis is warranted.

**Generally, very well written and error free.**

 “begs the question” does not mean “asks the question”.

I have read this submission. I believe that I have an appropriate level of expertise to confirm that it is of an acceptable scientific standard.
